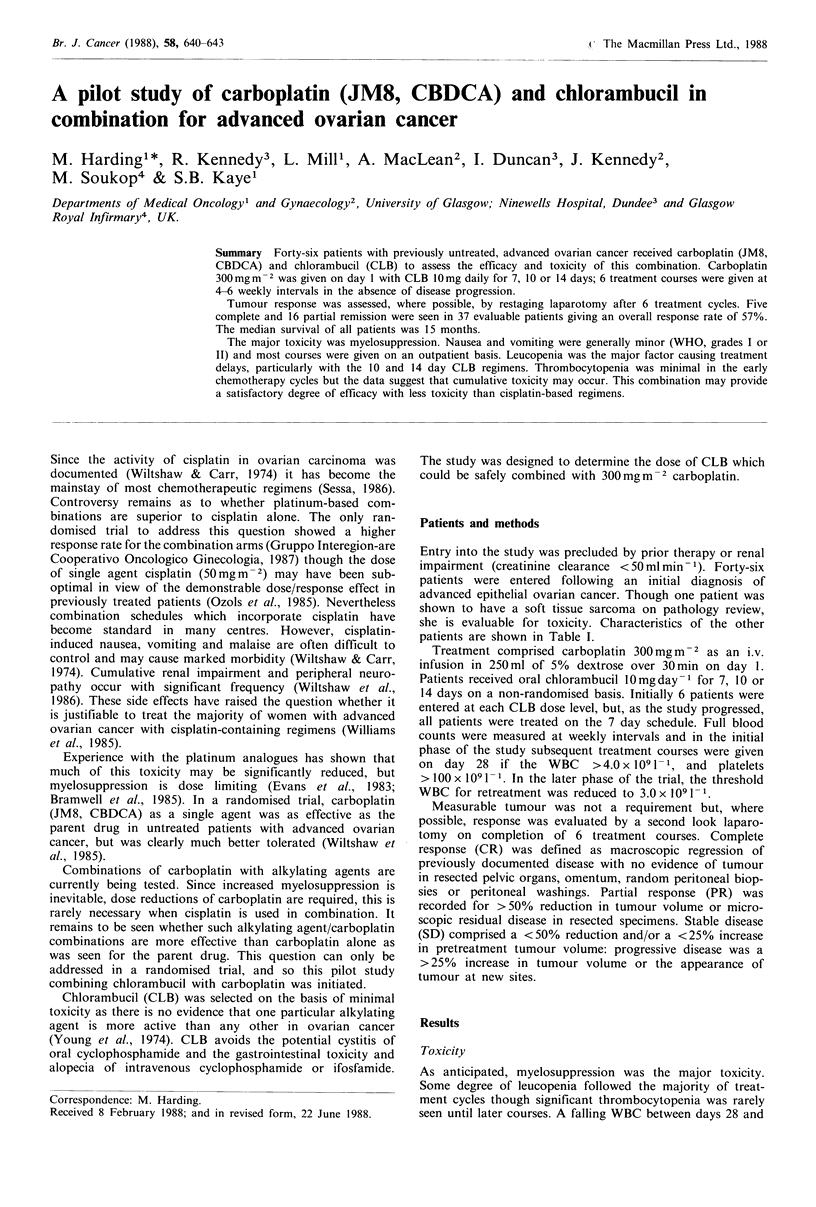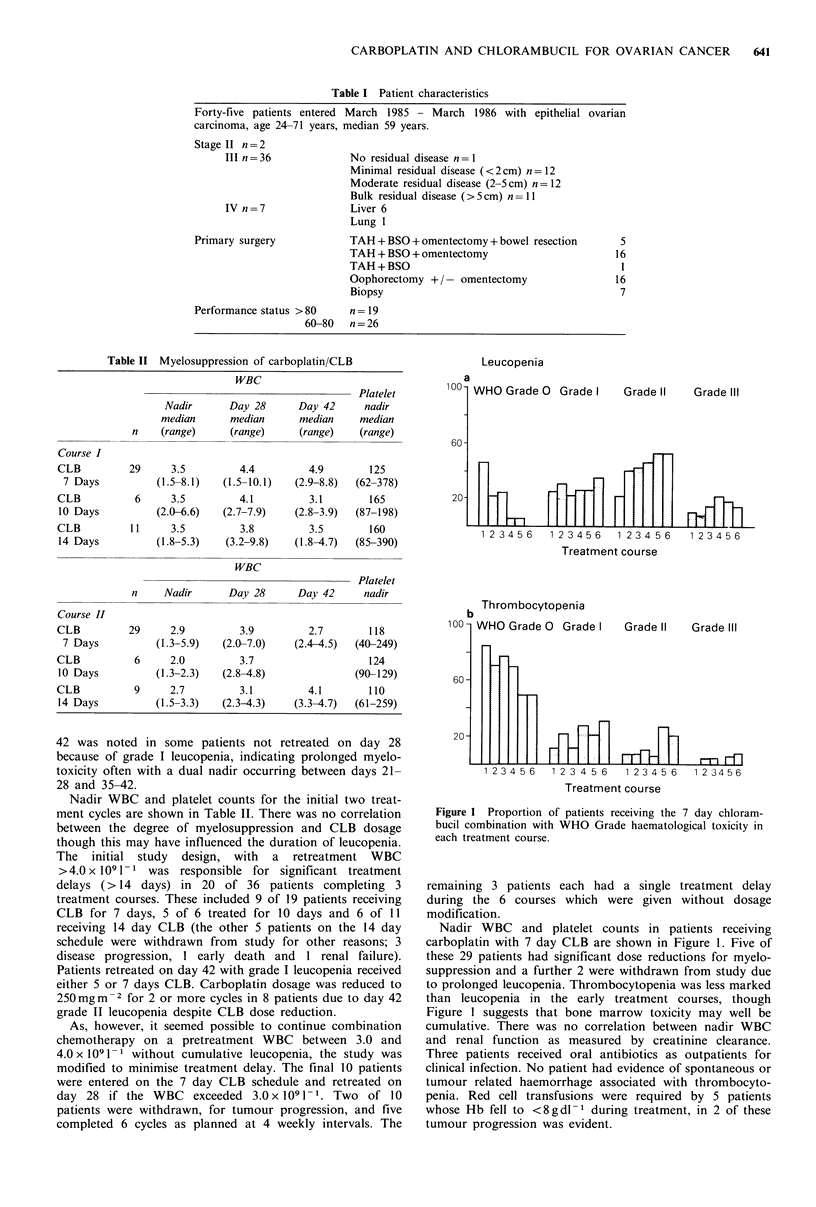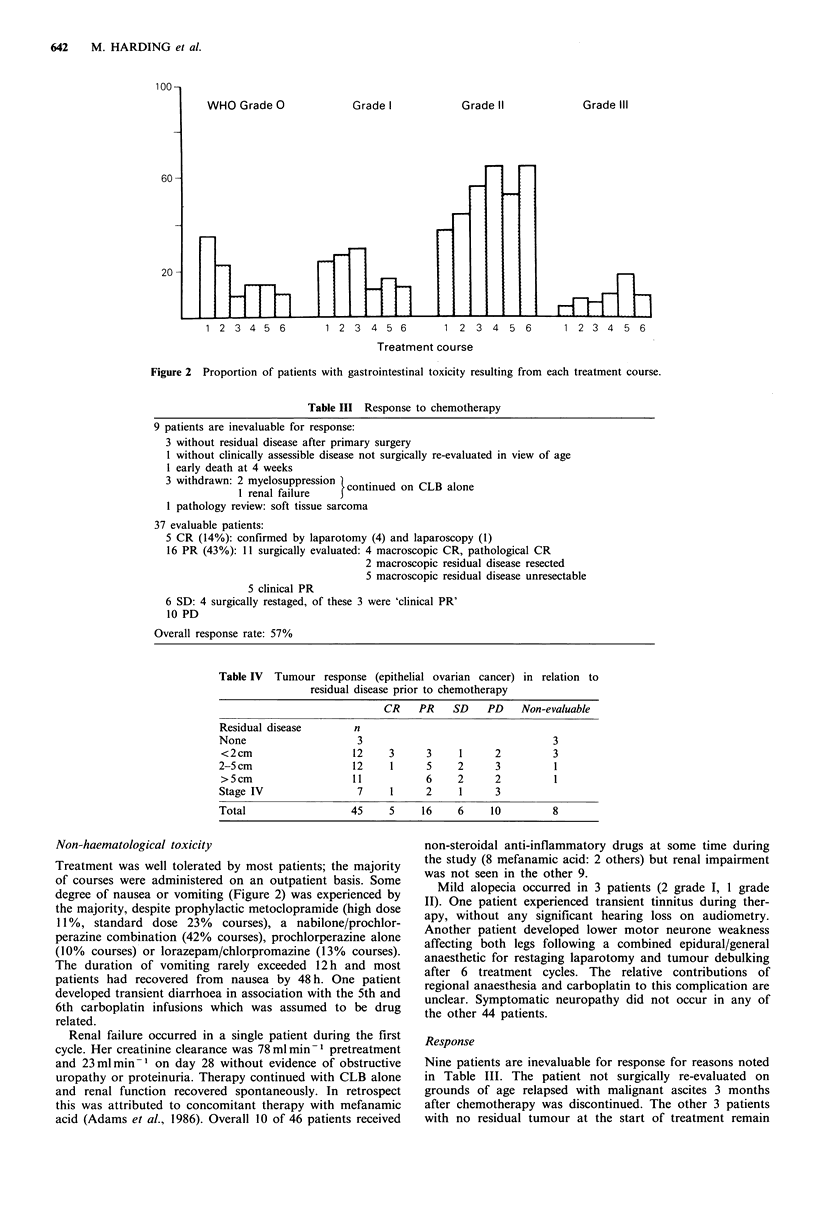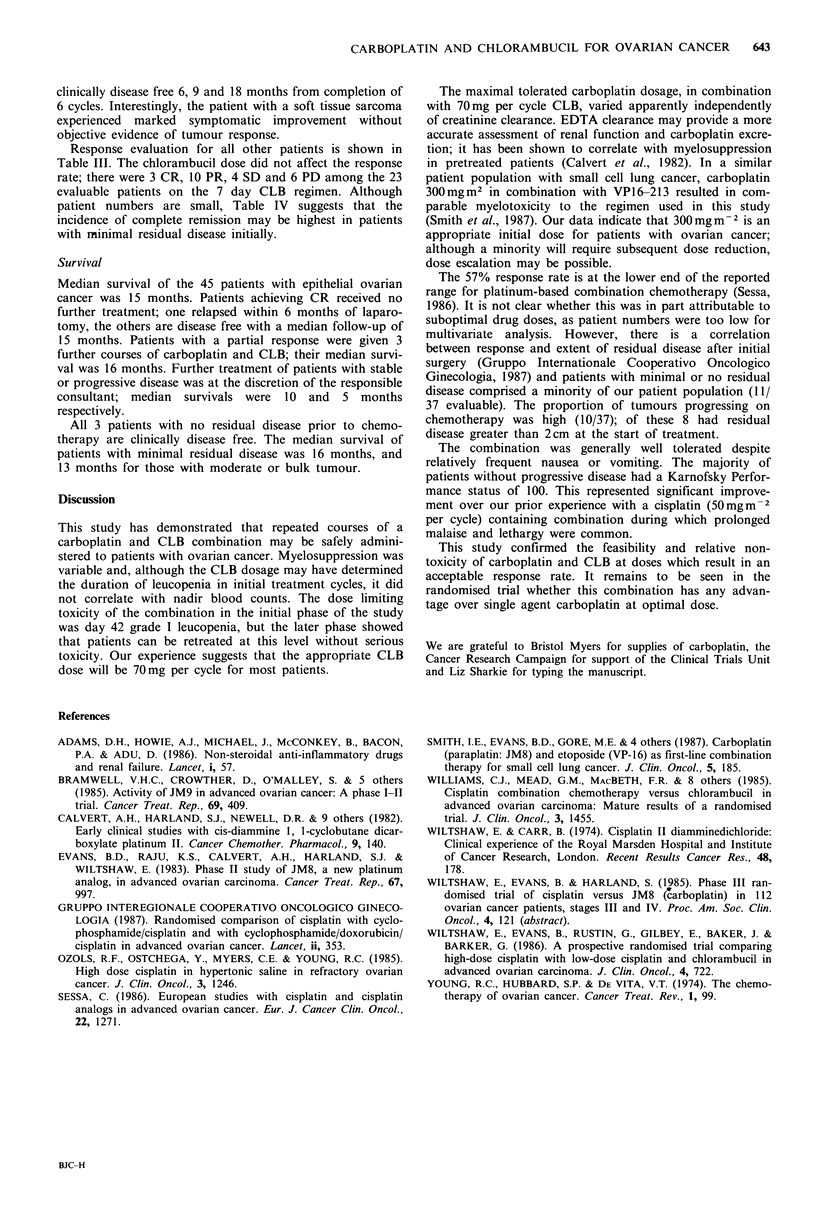# A pilot study of carboplatin (JM8, CBDCA) and chlorambucil in combination for advanced ovarian cancer.

**DOI:** 10.1038/bjc.1988.276

**Published:** 1988-11

**Authors:** M. Harding, R. Kennedy, L. Mill, A. MacLean, I. Duncan, J. Kennedy, M. Soukop, S. B. Kaye

**Affiliations:** Department of Medical Oncology, University of Glasgow, UK.

## Abstract

Forty-six patients with previously untreated, advanced ovarian cancer received carboplatin (JM8, CBDCA) and chlorambucil (CLB) to assess the efficacy and toxicity of this combination. Carboplatin 300 mg m-2 was given on day 1 with CLB 10 mg daily for 7, 10 or 14 days; 6 treatment courses were given at 4-6 weekly intervals in the absence of disease progression. Tumour response was assessed, where possible, by restaging laparotomy after 6 treatment cycles. Five complete and 16 partial remission were seen in 37 evaluable patients giving an overall response rate of 57%. The median survival of all patients was 15 months. The major toxicity was myelosuppression. Nausea and vomiting were generally minor (WHO, grades I or II) and most courses were given on an outpatient basis. Leucopenia was the major factor causing treatment delays, particularly with the 10 and 14 day CLB regimens. Thrombocytopenia was minimal in the early chemotherapy cycles but the data suggest that cumulative toxicity may occur. This combination may provide a satisfactory degree of efficacy with less toxicity than cisplatin-based regimens.


Br. J. Cancer (1988), 58, 640-643 © The Macmillan Press Ltd., 1988

### A pilot study of carboplatin (JM8, CBDCA) and chlorambucil in combination for advanced ovarian cancer

M. Harding[1]*, R. Kennedy[3], L. Mill[1], A. MacLean[2], I. Duncan[3], J. Kennedy[2], M. Soukop[4] & S.B. Kaye[1]

*Departments of Medical Oncology[1] and Gynaecology[2], University of Glasgow; Ninewells Hospital, Dundee[3] and Glasgow Royal Infirmary[4], UK.*

**Summary** Forty-six patients with previously untreated, advanced ovarian cancer received carboplatin (JM8, CBDCA) and chlorambucil (CLB) to assess the efficacy and toxicity of this combination. Carboplatin $300\,mg\,m^{-2}$ was given on day 1 with CLB 10 mg daily for 7, 10 or 14 days; 6 treatment courses were given at 4–6 weekly intervals in the absence of disease progression.

Tumour response was assessed, where possible, by restaging laparotomy after 6 treatment cycles. Five complete and 16 partial remission were seen in 37 evaluable patients giving an overall response rate of 57%. The median survival of all patients was 15 months.

The major toxicity was myelosuppression. Nausea and vomiting were generally minor (WHO, grades I or II) and most courses were given on an outpatient basis. Leucopenia was the major factor causing treatment delays, particularly with the 10 and 14 day CLB regimens. Thrombocytopenia was minimal in the early chemotherapy cycles but the data suggest that cumulative toxicity may occur. This combination may provide a satisfactory degree of efficacy with less toxicity than cisplatin-based regimens.

Since the activity of cisplatin in ovarian carcinoma was documented (Wiltshaw & Carr, 1974) it has become the mainstay of most chemotherapeutic regimens (Sessa, 1986). Controversy remains as to whether platinum-based combinations are superior to cisplatin alone. The only randomised trial to address this question showed a higher response rate for the combination arms (Gruppo Interegion-are Cooperativo Oncologico Ginecologia, 1987) though the dose of single agent cisplatin ($50\,mg\,m^{-2}$) may have been sub-optimal in view of the demonstrable dose/response effect in previously treated patients (Ozols *et al.*, 1985). Nevertheless combination schedules which incorporate cisplatin have become standard in many centres. However, cisplatin-induced nausea, vomiting and malaise are often difficult to control and may cause marked morbidity (Wiltshaw & Carr, 1974). Cumulative renal impairment and peripheral neuropathy occur with significant frequency (Wiltshaw *et al.*, 1986). These side effects have raised the question whether it is justifiable to treat the majority of women with advanced ovarian cancer with cisplatin-containing regimens (Williams *et al.*, 1985).

Experience with the platinum analogues has shown that much of this toxicity may be significantly reduced, but myelosuppression is dose limiting (Evans *et al.*, 1983; Bramwell *et al.*, 1985). In a randomised trial, carboplatin (JM8, CBDCA) as a single agent was as effective as the parent drug in untreated patients with advanced ovarian cancer, but was clearly much better tolerated (Wiltshaw *et al.*, 1985).

Combinations of carboplatin with alkylating agents are currently being tested. Since increased myelosuppression is inevitable, dose reductions of carboplatin are required, this is rarely necessary when cisplatin is used in combination. It remains to be seen whether such alkylating agent/carboplatin combinations are more effective than carboplatin alone as was seen for the parent drug. This question can only be addressed in a randomised trial, and so this pilot study combining chlorambucil with carboplatin was initiated.

Chlorambucil (CLB) was selected on the basis of minimal toxicity as there is no evidence that one particular alkylating agent is more active than any other in ovarian cancer (Young *et al.*, 1974). CLB avoids the potential cystitis of oral cyclophosphamide and the gastrointestinal toxicity and alopecia of intravenous cyclophosphamide or ifosfamide. The study was designed to determine the dose of CLB which could be safely combined with $300\,mg\,m^{-2}$ carboplatin.

Correspondence: M. Harding.
Received 8 February 1988; and in revised form, 22 June 1988.

### Patients and methods

Entry into the study was precluded by prior therapy or renal impairment (creatinine clearance $<50\,ml\,min^{-1}$). Forty-six patients were entered following an initial diagnosis of advanced epithelial ovarian cancer. Though one patient was shown to have a soft tissue sarcoma on pathology review, she is evaluable for toxicity. Characteristics of the other patients are shown in Table I.

Treatment comprised carboplatin $300\,mg\,m^{-2}$ as an i.v. infusion in 250 ml of 5% dextrose over 30 min on day 1. Patients received oral chlorambucil $10\,mg\,day^{-1}$ for 7, 10 or 14 days on a non-randomised basis. Initially 6 patients were entered at each CLB dose level, but, as the study progressed, all patients were treated on the 7 day schedule. Full blood counts were measured at weekly intervals and in the initial phase of the study subsequent treatment courses were given on day 28 if the WBC $>4.0\times10^{9}\,l^{-1}$, and platelets $>100\times10^{9}\,l^{-1}$. In the later phase of the trial, the threshold WBC for retreatment was reduced to $3.0\times10^{9}\,l^{-1}$.

Measurable tumour was not a requirement but, where possible, response was evaluated by a second look laparotomy on completion of 6 treatment courses. Complete response (CR) was defined as macroscopic regression of previously documented disease with no evidence of tumour in resected pelvic organs, omentum, random peritoneal biopsies or peritoneal washings. Partial response (PR) was recorded for >50% reduction in tumour volume or microscopic residual disease in resected specimens. Stable disease (SD) comprised a <50% reduction and/or a <25% increase in pretreatment tumour volume: progressive disease was a >25% increase in tumour volume or the appearance of tumour at new sites.

### Results

### *Toxicity*

As anticipated, myelosuppression was the major toxicity. Some degree of leucopenia followed the majority of treatment cycles though significant thrombocytopenia was rarely seen until later courses. A falling WBC between days 28 and

**Table I** Patient characteristics

Forty-five patients entered March 1985 – March 1986 with epithelial ovarian carcinoma, age 24–71 years, median 59 years.

| | | |
|---|---|---|
| Stage II $n=2$ | | |
| III $n=36$ | No residual disease $n=1$ | |
| | Minimal residual disease (<2 cm) $n=12$ | |
| | Moderate residual disease (2–5 cm) $n=12$ | |
| | Bulk residual disease (>5 cm) $n=11$ | |
| IV $n=7$ | Liver 6 | |
| | Lung 1 | |
| Primary surgery | TAH + BSO + omentectomy + bowel resection | 5 |
| | TAH + BSO + omentectomy | 16 |
| | TAH + BSO | 1 |
| | Oophorectomy +/− omentectomy | 16 |
| | Biopsy | 7 |
| Performance status >80 | $n=19$ | |
| 60–80 | $n=26$ | |

**Table II** Myelosuppression of carboplatin/CLB

| | | *WBC* | | | |
|---|---|---|---|---|---|
| | *n* | *Nadir median (range)* | *Day 28 median (range)* | *Day 42 median (range)* | *Platelet nadir median (range)* |
| *Course I* | | | | | |
| CLB 7 Days | 29 | 3.5 (1.5–8.1) | 4.4 (1.5–10.1) | 4.9 (2.9–8.8) | 125 (62–378) |
| CLB 10 Days | 6 | 3.5 (2.0–6.6) | 4.1 (2.7–7.9) | 3.1 (2.8–3.9) | 165 (87–198) |
| CLB 14 Days | 11 | 3.5 (1.8–5.3) | 3.8 (3.2–9.8) | 3.5 (1.8–4.7) | 160 (85–390) |

| | | *WBC* | | | |
|---|---|---|---|---|---|
| | *n* | *Nadir* | *Day 28* | *Day 42* | *Platelet nadir* |
| *Course II* | | | | | |
| CLB 7 Days | 29 | 2.9 (1.3–5.9) | 3.9 (2.0–7.0) | 2.7 (2.4–4.5) | 118 (40–249) |
| CLB 10 Days | 6 | 2.0 (1.3–2.3) | 3.7 (2.8–4.8) | | 124 (90–129) |
| CLB 14 Days | 9 | 2.7 (1.5–3.3) | 3.1 (2.3–4.3) | 4.1 (3.3–4.7) | 110 (61–259) |

42 was noted in some patients not retreated on day 28 because of grade I leucopenia, indicating prolonged myelotoxicity often with a dual nadir occurring between days 21–28 and 35–42.

Nadir WBC and platelet counts for the initial two treatment cycles are shown in Table II. There was no correlation between the degree of myelosuppression and CLB dosage though this may have influenced the duration of leucopenia. The initial study design, with a retreatment WBC $>4.0\times10^{9}\,l^{-1}$ was responsible for significant treatment delays (>14 days) in 20 of 36 patients completing 3 treatment courses. These included 9 of 19 patients receiving CLB for 7 days, 5 of 6 treated for 10 days and 6 of 11 receiving 14 day CLB (the other 5 patients on the 14 day schedule were withdrawn from study for other reasons; 3 disease progression, 1 early death and 1 renal failure). Patients retreated on day 42 with grade I leucopenia received either 5 or 7 days CLB. Carboplatin dosage was reduced to $250\,mg\,m^{-2}$ for 2 or more cycles in 8 patients due to day 42 grade II leucopenia despite CLB dose reduction.

As, however, it seemed possible to continue combination chemotherapy on a pretreatment WBC between 3.0 and $4.0\times10^{9}\,l^{-1}$ without cumulative leucopenia, the study was modified to minimise treatment delay. The final 10 patients were entered on the 7 day CLB schedule and retreated on day 28 if the WBC exceeded $3.0\times10^{9}\,l^{-1}$. Two of 10 patients were withdrawn, for tumour progression, and five completed 6 cycles as planned at 4 weekly intervals. The remaining 3 patients each had a single treatment delay during the 6 courses which were given without dosage modification.

**Figure 1** Proportion of patients receiving the 7 day chlorambucil combination with WHO Grade haematological toxicity in each treatment course.

Nadir WBC and platelet counts in patients receiving carboplatin with 7 day CLB are shown in Figure 1. Five of these 29 patients had significant dose reductions for myelosuppression and a further 2 were withdrawn from study due to prolonged leucopenia. Thrombocytopenia was less marked than leucopenia in the early treatment courses, though Figure 1 suggests that bone marrow toxicity may well be cumulative. There was no correlation between nadir WBC and renal function as measured by creatinine clearance. Three patients received oral antibiotics as outpatients for clinical infection. No patient had evidence of spontaneous or tumour related haemorrhage associated with thrombocytopenia. Red cell transfusions were required by 5 patients whose Hb fell to $<8\,g\,dl^{-1}$ during treatment, in 2 of these tumour progression was evident.

**Figure 2** Proportion of patients with gastrointestinal toxicity resulting from each treatment course.

**Table III** Response to chemotherapy

9 patients are inevaluable for response:
- 3 without residual disease after primary surgery
- 1 without clinically assessible disease not surgically re-evaluated in view of age
- 1 early death at 4 weeks
- 3 withdrawn: 2 myelosuppression, 1 renal failure } continued on CLB alone
- 1 pathology review: soft tissue sarcoma

37 evaluable patients:
- 5 CR (14%): confirmed by laparotomy (4) and laparoscopy (1)
- 16 PR (43%): 11 surgically evaluated: 4 macroscopic CR, pathological CR
  - 2 macroscopic residual disease resected
  - 5 macroscopic residual disease unresectable
  - 5 clinical PR
- 6 SD: 4 surgically restaged, of these 3 were 'clinical PR'
- 10 PD

Overall response rate: 57%

**Table IV** Tumour response (epithelial ovarian cancer) in relation to residual disease prior to chemotherapy

| | | *CR* | *PR* | *SD* | *PD* | *Non-evaluable* |
|---|---|---|---|---|---|---|
| Residual disease | *n* | | | | | |
| None | 3 | | | | | 3 |
| <2 cm | 12 | 3 | 3 | 1 | 2 | 3 |
| 2–5 cm | 12 | 1 | 5 | 2 | 3 | 1 |
| >5 cm | 11 | | 6 | 2 | 2 | 1 |
| Stage IV | 7 | 1 | 2 | 1 | 3 | |
| Total | 45 | 5 | 16 | 6 | 10 | 8 |

*Non-haematological toxicity*

Treatment was well tolerated by most patients; the majority of courses were administered on an outpatient basis. Some degree of nausea or vomiting (Figure 2) was experienced by the majority, despite prophylactic metoclopramide (high dose 11%, standard dose 23% courses), a nabilone/prochlorperazine combination (42% courses), prochlorperazine alone (10% courses) or lorazepam/chlorpromazine (13% courses). The duration of vomiting rarely exceeded 12 h and most patients had recovered from nausea by 48 h. One patient developed transient diarrhoea in association with the 5th and 6th carboplatin infusions which was assumed to be drug related.

Renal failure occurred in a single patient during the first cycle. Her creatinine clearance was 78 ml min$^{-1}$ pretreatment and 23 ml min$^{-1}$ on day 28 without evidence of obstructive uropathy or proteinuria. Therapy continued with CLB alone and renal function recovered spontaneously. In retrospect this was attributed to concomitant therapy with mefanamic acid (Adams *et al.*, 1986). Overall 10 of 46 patients received non-steroidal anti-inflammatory drugs at some time during the study (8 mefanamic acid: 2 others) but renal impairment was not seen in the other 9.

Mild alopecia occurred in 3 patients (2 grade I, 1 grade II). One patient experienced transient tinnitus during therapy, without any significant hearing loss on audiometry. Another patient developed lower motor neurone weakness affecting both legs following a combined epidural/general anaesthetic for restaging laparotomy and tumour debulking after 6 treatment cycles. The relative contributions of regional anaesthesia and carboplatin to this complication are unclear. Symptomatic neuropathy did not occur in any of the other 44 patients.

*Response*

Nine patients are inevaluable for response for reasons noted in Table III. The patient not surgically re-evaluated on grounds of age relapsed with malignant ascites 3 months after chemotherapy was discontinued. The other 3 patients with no residual tumour at the start of treatment remain clinically disease free 6, 9 and 18 months from completion of 6 cycles. Interestingly, the patient with a soft tissue sarcoma experienced marked symptomatic improvement without objective evidence of tumour response.

Response evaluation for all other patients is shown in Table III. The chlorambucil dose did not affect the response rate; there were 3 CR, 10 PR, 4 SD and 6 PD among the 23 evaluable patients on the 7 day CLB regimen. Although patient numbers are small, Table IV suggests that the incidence of complete remission may be highest in patients with minimal residual disease initially.

*Survival*

Median survival of the 45 patients with epithelial ovarian cancer was 15 months. Patients achieving CR received no further treatment; one relapsed within 6 months of laparotomy, the others are disease free with a median follow-up of 15 months. Patients with a partial response were given 3 further courses of carboplatin and CLB; their median survival was 16 months. Further treatment of patients with stable or progressive disease was at the discretion of the responsible consultant; median survivals were 10 and 5 months respectively.

All 3 patients with no residual disease prior to chemotherapy are clinically disease free. The median survival of patients with minimal residual disease was 16 months, and 13 months for those with moderate or bulk tumour.

### Discussion

This study has demonstrated that repeated courses of a carboplatin and CLB combination may be safely administered to patients with ovarian cancer. Myelosuppression was variable and, although the CLB dosage may have determined the duration of leucopenia in initial treatment cycles, it did not correlate with nadir blood counts. The dose limiting toxicity of the combination in the initial phase of the study was day 42 grade I leucopenia, but the later phase showed that patients can be retreated at this level without serious toxicity. Our experience suggests that the appropriate CLB dose will be 70 mg per cycle for most patients.

The maximal tolerated carboplatin dosage, in combination with 70 mg per cycle CLB, varied apparently independently of creatinine clearance. EDTA clearance may provide a more accurate assessment of renal function and carboplatin excretion; it has been shown to correlate with myelosuppression in pretreated patients (Calvert *et al.*, 1982). In a similar patient population with small cell lung cancer, carboplatin 300 mg m$^2$ in combination with VP16–213 resulted in comparable myelotoxicity to the regimen used in this study (Smith *et al.*, 1987). Our data indicate that 300 mg m$^{-2}$ is an appropriate initial dose for patients with ovarian cancer; although a minority will require subsequent dose reduction, dose escalation may be possible.

The 57% response rate is at the lower end of the reported range for platinum-based combination chemotherapy (Sessa, 1986). It is not clear whether this was in part attributable to suboptimal drug doses, as patient numbers were too low for multivariate analysis. However, there is a correlation between response and extent of residual disease after initial surgery (Gruppo Internationale Cooperativo Oncologico Ginecologia, 1987) and patients with minimal or no residual disease comprised a minority of our patient population (11/37 evaluable). The proportion of tumours progressing on chemotherapy was high (10/37); of these 8 had residual disease greater than 2 cm at the start of treatment.

The combination was generally well tolerated despite relatively frequent nausea or vomiting. The majority of patients without progressive disease had a Karnofsky Performance status of 100. This represented significant improvement over our prior experience with a cisplatin (50 mg m$^{-2}$ per cycle) containing combination during which prolonged malaise and lethargy were common.

This study confirmed the feasibility and relative non-toxicity of carboplatin and CLB at doses which result in an acceptable response rate. It remains to be seen in the randomised trial whether this combination has any advantage over single agent carboplatin at optimal dose.

We are grateful to Bristol Myers for supplies of carboplatin, the Cancer Research Campaign for support of the Clinical Trials Unit and Liz Sharkie for typing the manuscript.

### References

ADAMS, D.H., HOWIE, A.J., MICHAEL, J., McCONKEY, B., BACON, P.A. & ADU, D. (1986). Non-steroidal anti-inflammatory drugs and renal failure. *Lancet*, **i**, 57.

BRAMWELL, V.H.C., CROWTHER, D., O'MALLEY, S. & 5 others (1985). Activity of JM9 in advanced ovarian cancer: A phase I–II trial. *Cancer Treat. Rep.*, **69**, 409.

CALVERT, A.H., HARLAND, S.J., NEWELL, D.R. & 9 others (1982). Early clinical studies with cis-diammine 1, 1-cyclobutane dicarboxylate platinum II. *Cancer Chemother. Pharmacol.*, **9**, 140.

EVANS, B.D., RAJU, K.S., CALVERT, A.H., HARLAND, S.J. & WILTSHAW, E. (1983). Phase II study of JM8, a new platinum analog, in advanced ovarian carcinoma. *Cancer Treat. Rep.*, **67**, 997.

GRUPPO INTEREGIONALE COOPERATIVO ONCOLOGICO GINECOLOGIA (1987). Randomised comparison of cisplatin with cyclophosphamide/cisplatin and with cyclophosphamide/doxorubicin/cisplatin in advanced ovarian cancer. *Lancet*, **ii**, 353.

OZOLS, R.F., OSTCHEGA, Y., MYERS, C.E. & YOUNG, R.C. (1985). High dose cisplatin in hypertonic saline in refractory ovarian cancer. *J. Clin. Oncol.*, **3**, 1246.

SESSA, C. (1986). European studies with cisplatin and cisplatin analogs in advanced ovarian cancer. *Eur. J. Cancer Clin. Oncol.*, **22**, 1271.

SMITH, I.E., EVANS, B.D., GORE, M.E. & 4 others (1987). Carboplatin (paraplatin: JM8) and etoposide (VP-16) as first-line combination therapy for small cell lung cancer. *J. Clin. Oncol.*, **5**, 185.

WILLIAMS, C.J., MEAD, G.M., MacBETH, F.R. & 8 others (1985). Cisplatin combination chemotherapy versus chlorambucil in advanced ovarian carcinoma: Mature results of a randomised trial. *J. Clin. Oncol.*, **3**, 1455.

WILTSHAW, E. & CARR, B. (1974). Cisplatin II diamminedichloride: Clinical experience of the Royal Marsden Hospital and Institute of Cancer Research, London. *Recent Results Cancer Res.*, **48**, 178.

WILTSHAW, E., EVANS, B. & HARLAND, S. (1985). Phase III randomised trial of cisplatin versus JM8 (carboplatin) in 112 ovarian cancer patients, stages III and IV. *Proc. Am. Soc. Clin. Oncol.*, **4**, 121 (*abstract*).

WILTSHAW, E., EVANS, B., RUSTIN, G., GILBEY, E., BAKER, J. & BARKER, G. (1986). A prospective randomised trial comparing high-dose cisplatin with low-dose cisplatin and chlorambucil in advanced ovarian carcinoma. *J. Clin. Oncol.*, **4**, 722.

YOUNG, R.C., HUBBARD, S.P. & De VITA, V.T. (1974). The chemotherapy of ovarian cancer. *Cancer Treat. Rev.*, **1**, 99.